# A perspective on euthanasia

**DOI:** 10.1038/sj.bjc.6603365

**Published:** 2006-10-17

**Authors:** D Oliver

**Affiliations:** 1 Wisdom Hospice, High Bank, Rochester, Kent ME1 2NU, UK; 2 Kent Institute of Medicine and Health Sciences, University of Kent, Canterbury, UK

Throughout the world there are discussions regarding end-of-life issues and the assistance of dying by medical and other professionals. Cancer patients do ask about end-of-life issues and if health care professionals are open, many will ask about assisted dying – in Oregon, the rate of death from physician-assisted suicide (PAS) for cancer patients was 61/10 000 deaths compared with the overall rate of 8.8/10 000 deaths (Hedberg *et al*, 2003). Oncologists may become involved in these discussions and may face problems having made decisions – in the US, a survey of physicians showed that 24% of doctors who had assisted in euthanasia or PAS, usually outside the legal framework, regretted their decision on reflection (Emanuel *et al*, 1998). The issue of assisted dying will affect all of us and we all need to be clear of our own views and within our own ethical standpoints. If the legal system does allow assisted death, this becomes even more real and more important.

These discussions are complicated as there is confusion about the exact terminology used and it is essential that these are clarified:

- Euthanasia is when a doctor intentionally kills a person by the administration of drugs at that person's voluntary and competent request (Materstvedt *et al*, 2003)
- PAS is when a doctor intentionally helps a person to commit suicide by providing drugs for self-administration at that person's voluntary and competent request (Materstvedt *et al*, 2003).
- Nonvoluntary termination of life is the administration of medication to end the life of a patient who is unable to give competent request for this at the time of administration.
- The withholding or withdrawal of treatment, such as parenteral fluids, at the request of the patient is not euthanasia.
- The administration of medication to control symptoms, such as increased analgesia or sedation for a confused and agitated patient, is not euthanasia as the intention is the management of the symptom rather than the intention to kill the patient – the principle of double effect.

All these issues are, on occasions, confused and there tend to be firmly held views, with a reluctance to negotiate, on either side of the debate. However, there is the need for serious consideration of these issues so that health care professionals are able to discuss end-of-life issues appropriately with patients and families.

There are several countries where assisted dying is allowed:

- In the Netherlands both euthanasia and PAS are permitted by law, provided clearly defined protocols are followed
- In Oregon, state PAS is permitted. Up to 50 people take this option every year, although many more people ask for the medication to be prescribed, and then never take it
- In Belgium, euthanasia is permitted by law, with clear guidelines that need to be followed
- In Switzerland, assisting suicide is permitted, under legislation dating back over 600 years. Patients, including visitors from other countries, may be helped by members of the organization Dignitas to end their lives,
- For a short period of time, euthanasia and PAS were permitted in the Northern Territory of Australia and seven people ended their lives in this way, before the Australian Federal Government overturned the law.

In the UK, there have been several attempts to change the law. Recently, Lord Joffe introduced an Assisted Dying Bill into the House of Lords, with the aim of allowing PAS. This was defeated but further bills are expected to be introduced in the future. Opinion polls of the general public show that there is widespread approval for a change in the law, whereas the medical profession has been more circumspect and recent polls of members of two Royal Colleges in the UK (General Practitioners and Physicians in England) have shown that only a minority of doctors support a change.

The arguments for assisted dying centre primarily on the support of a person's autonomous decision to end their life. It is proposed that if someone, usually, but not exclusively, a person near the end-of-life, has unbearable symptoms or distress and feels that their quality of life is poor, they should be able to ask for an assisted death. However, for any autonomous decision the person needs to be adequately informed and must be able to take the competent decision without coercion. These factors may not always be easily assessed and even in the Netherlands only a proportion – about one-third– of people requesting euthanasia receive an assisted death. The physician may be unclear as to whether alternative treatments are available and have been considered or whether the decision has not been adequately informed (ten Have and Welie, 2005). Evidence from the Netherlands has shown that doctors still exercise power over patients, and by refusing their request they are restricting their exercise of their autonomy. It has been argued that there is even greater medicalisation of medically procured deaths (ten Have and Welie, 2005).

All these areas are hotly disputed. It is, however, important to hear the request and the feelings behind it. A request for death may be an expression of fear, of being ‘kept alive’ by technological treatments, or an expression of depression, which could respond to treatment. More rarely it may be an expression of despair and loss, or fear of loss, of dignity. The evidence from Oregon is that the majority of people requesting PAS are white, male, more highly educated and often in managerial roles, who are finding the reduction in control of their lives difficult and are looking for control over their deaths (Ganzini *et al*, 2000). This is a small group for whom there may be no easy way of reducing their distress and anxieties. However, whether this justifies assisted death being made more widely available, with the risks to the more vulnerable, is debatable. Once death is seen as a ‘moral good’ – when death is considered to be in the patient's best interest as the distress is untenable and the quality of life unbearable – the care of dying people changes dramatically. Suffering is no longer seen as a normal part of our humanity and living, and death is seen as a preferable action to coping and trying to alleviate the suffering.

The very ill are often vulnerable. Someone whose condition is deteriorating and is coming to the end of their life may feel that death is an easier option – to eliminate the risk of pain, to stop the burden on the family, or to resolve their depression. In the Netherlands, it is now possible to receive assisted death for severe depression or even feelings of ‘meaninglessness of life’ in old age (ten Have and Welie, 2005). Disabled or abnormal infants have been assisted in dying – when no competent decision could be given. Although there is little evidence from the official figures of an increase in the numbers of assisted deaths in the Netherlands, there is however, concern that the proportion of cases reported to the authorities is reducing and many go unreported.

Palliative care can offer much to patients and their families, involving the physical, psychosocial and spiritual aspects of care of both patient and family. It becomes even more imperative to ensure that patients are able to access care from a specialist multidisciplinary team. Most requests for assisted dying can be resolved with good communication, understanding and symptom control. There may still be a small number of people who find the fears or reality of indignity too much and who may then ask for assisted dying. This is a decision that will be taken by society in general, who need to be adequately informed of the issues, and all health care professionals need to consider how to respond both individually and collectively.
